# Bio-SCoRes: A Smorgasbord Architecture for Coreference Resolution in Biomedical Text

**DOI:** 10.1371/journal.pone.0148538

**Published:** 2016-03-02

**Authors:** Halil Kilicoglu, Dina Demner-Fushman

**Affiliations:** Lister Hill National Center for Biomedical Communications, National Library of Medicine, National Institutes of Health, Bethesda, MD, United States of America; Garvan Institute of Medical Research, AUSTRALIA

## Abstract

Coreference resolution is one of the fundamental and challenging tasks in natural language processing. Resolving coreference successfully can have a significant positive effect on downstream natural language processing tasks, such as information extraction and question answering. The importance of coreference resolution for biomedical text analysis applications has increasingly been acknowledged. One of the difficulties in coreference resolution stems from the fact that distinct types of coreference (e.g., anaphora, appositive) are expressed with a variety of lexical and syntactic means (e.g., personal pronouns, definite noun phrases), and that resolution of each combination often requires a different approach. In the biomedical domain, it is common for coreference annotation and resolution efforts to focus on specific subcategories of coreference deemed important for the downstream task. In the current work, we aim to address some of these concerns regarding coreference resolution in biomedical text. We propose a general, modular framework underpinned by a *smorgasbord* architecture (Bio-SCoRes), which incorporates a variety of coreference types, their mentions and allows fine-grained specification of resolution strategies to resolve coreference of distinct coreference type-mention pairs. For development and evaluation, we used a corpus of structured drug labels annotated with fine-grained coreference information. In addition, we evaluated our approach on two other corpora (i2b2/VA discharge summaries and protein coreference dataset) to investigate its generality and ease of adaptation to other biomedical text types. Our results demonstrate the usefulness of our novel smorgasbord architecture. The specific pipelines based on the architecture perform successfully in linking coreferential mention pairs, while we find that recognition of full mention clusters is more challenging. The corpus of structured drug labels (SPL) as well as the components of Bio-SCoRes and some of the pipelines based on it are publicly available at https://github.com/kilicogluh/Bio-SCoRes. We believe that Bio-SCoRes can serve as a strong and extensible baseline system for coreference resolution of biomedical text.

## Introduction

Coreference is defined as the relation between linguistic expressions that are referring to the same real-world entity [1]. Coreference resolution is the task of recognizing these expressions in text and linking or chaining them. It is one of the fundamental tasks in natural language processing and can be critical for many downstream tasks, such as relation extraction, automatic summarization, textual entailment, and question answering. Coreference is generally trivial for humans to resolve and it occurs frequently in all types of text. For instance, consider the simple example below [2]:

(1) John_X_ is {a linguist}_X_. People_Y_ are nervous around John_X_, because he_X_ always corrects their_Y_ grammar.

The mentions with the same subscripts corefer. Often, two major types of coreference relations are distinguished: identity and appositive. The coreference relation between *John* and *he* and the one between *People* and *their* are relations of identity (identity); in other words, the expressions refer to the same entities in the real world. Both coreference relations are signaled with pronouns (personal and possessive, respectively) and are instances of *anaphora*, since the coreferential mentions (*anaphor*s) point back to previously mentioned items (*antecedent*s) in text. On the other hand, the relation between *John* and *a linguist* is an attribute relation (appositive). The attribute relation in this instance is indicated by using an indefinite noun phrase (*a linguist*) in a copular construction. This type of copular coreference is sometimes referred to as *predicate nominative* [3]. In appositive relations, coreferential mention is called an *attribute* and the referent a *head* [2]. Coreference can be represented as a pairwise relation or as a chain. In the example above, three pairwise relations (*{John,a linguist}, {John,he}, {People,their}*) and two chains (*{John,a linguist,he}* and *{People,their}*) can be distinguished.

Coreference is common in all types of biomedical text, as well. Consider the example below, taken from a drug label in DailyMed [4]:

(2) Since amiodarone is a substrate for {CYP3A and CYP2C8}_X_, drugs/substances that inhibit {these isoenzymes}_X_ may decrease the metabolism ….

In this example, the anaphor is a demonstrative noun phrase (*these isoenzymes*) and its antecedents are *CYP3A* and *CYP2C8*, constituting an instance of anaphora. This anaphora instance also exemplifies a set-membership relation, since *CYP3A* and *CYP2C8* are members of the *isoenzyme* set. Resolving this coreference instance would allow us to capture the following drug interactions mentioned in the sentence:

- Inhibitors of CYP3A-POTENTIATE-Amiodarone
- Inhibitors of CYP2C8-POTENTIATE-Amiodarone

Although research in coreference resolution in computational linguistics dates back to at least 1970s (e.g., Hobbs [5]), it is a difficult problem that remains far from being solved. This can be attributed to several factors:

- Coreference is a complex, discourse-level phenomenon, and its resolution needs to take into account the entire text.
- There are several distinct subtypes of coreference, such as anaphora, cataphora, and bridging. Each can be expressed using a variety of lexical/syntactic means, as we have seen in the examples above; each combination requiring, sometimes significantly, different processing.
- All levels of linguistic information, from morphosyntactic to semantic and discourse-level, contribute to coreference resolution and this requires high-quality natural language processing tools that can address these levels of information accurately.
- Corpus annotation for coreference has been notoriously difficult, partly due to cognitive burden it places on the annotator and partly due to terminological confusion [6], often leading to low consistency in annotation [2]. The consequence of this has been the development of several annotation schemes addressing some aspects of coreference, but not others. For example, the pioneering MUC7 dataset [7] considered only identity relations. In the more recent OntoNotes corpus [8], predicate nominatives are not annotated, while explicit appositive constructions which have similar semantic consequences are (e.g., *John, a linguist* is annotated, while *John is a linguist* is not).
- The evaluation of coreference resolution systems has been controversial. Several evaluation metrics have been proposed [9–12], their results sometimes varying significantly. Pradhan et al. [2] have argued that, in the absence of a specific application, an objective measure of coreference resolution performance is difficult to establish.

Despite these challenges, recent years have seen important advances in this area of research. In the general domain, OntoNotes [8] has emerged as the standard corpus and the annotation scheme and CoNLL shared tasks based on this corpus have provided the platform for evaluation [2, 13]. New evaluation metrics have also been proposed [14].

In biomedical natural language processing (bioNLP), research on coreference resolution is relatively recent. BioNLP is an applied discipline and this is reflected in the mostly pragmatic manner coreference resolution task is approached within the community. It is generally considered a supporting task to more critical applications, such as event extraction or clinical text processing. Most of the early work focused on coreference resolution of specific entity types in biomedical literature, such as bio-entities (chemicals, genes, cells, etc.) [15] or proteins [16, 17]. Corpora have been made available, such as the GENIA protein coreference dataset, which has been used for the BioNLP 2011 supporting shared task on coreference resolution [18]. Coreference in clinical narratives has also been studied [1] and an i2b2/VA shared task on coreference resolution has been organized [19]. There have been attempts to use existing general domain coreference resolution tools (e.g., [20]) on clinical text and biomedical literature; however, the results reported have been generally poor [21, 22], suggesting that biomedical coreference resolution may be a more challenging problem and semantic constraints less utilized in the general domain can provide useful basis for coreference resolution.

In this paper, we focus on biomedical coreference resolution. Our goal has been to develop a modular, flexible coreference resolution framework that can accommodate a wide range of coreference types and expressions, while maintaining generality and extensibility. Towards this goal, we propose a smorgasbord architecture, based on the notion of *resolution strategies*. Each strategy essentially addresses a coreference type/mention type pair (e.g., anaphora expressed with definite noun phrases) and attempts to resolve instances of this specific combination. A strategy consists of a set of mechanisms for filtering mentions and their candidate referents, for measuring compatibility between a mention and a candidate referent, and for determining the best referent in a set of compatible candidates. The system incorporates a variety of methods for each of these steps, and we formulate coreference resolution as the task of selecting the appropriate methods for each step and combining them to reach desired performance on a given corpus or for a downstream task, analogous to selecting dishes from a smorgasbord. The core of the framework is designed to be corpus-agnostic, in the sense that additional methods can be plugged in to address corpus-specific concerns, and pre- and post-processing steps can be used to tailor input and output for specific tasks.

The work reported here extends and consolidates our previous work on coreference resolution in consumer health questions [23] and drug labels [24]. To aid and inform system development, we annotated a corpus of structured drug labels (SPLs) using a fine-grained coreference annotation scheme and a pairwise relation representation. The corpus focuses on drug/substance coreference, due to our interest in drug information extraction, specifically drug-drug interactions. To show the generality of our approach, we evaluated the system on two other biomedical corpora annotated for coreference: the GENIA protein coreference dataset [17] and i2b2/VA clinical coreference dataset [19], the former using pairwise relations and the latter using coreference chains (clusters). Our results show that our framework, in its current state, can address coreference in different types of biomedical text to varying degrees. The smorgasbord architecture provides the means for a strong, robust baseline, with the incorporated resolution mechanisms, while also allowing addition of new mechanisms. While the system performs better than the state-of-the-art results on biomedical literature, we find that there is room for improvement for coreference resolution in clinical reports. On the other hand, on SPLs, the system performs significantly better than the baseline, in the absence of a comparable system.

To avoid terminological confusion, it should be noted that coreferring expressions (e.g., anaphor and antecedent) are sometimes collectively called *markables* [7], while others make a distinction between the referring mention and the referent; for example, in the ACE corpus [25], the referring mentions are simply called mentions, and the referents are called entities. In this work, we will generally make the distinction between the referring mention and the referent, and use specific terms, such as anaphor or antecedent, when possible. When this is not relevant, we will use the terms *mention* and *referent*. We will also refer to specific coreference type under discussion (e.g., *anaphora*), when relevant, rather than using the generic term, coreference.

## Related Work

In the general English domain, early coreference resolution systems often focused on pronominal coreference using rule-based approaches [5, 26, 27]. These approaches relied on syntactic structure as well as discourse constraints, such as those predicted by the Centering Theory [28], which investigates the interaction of local coherence with the choice of linguistic expressions. With the availability of annotated corpora, such as MUC7 [7] and ACE [25], these approaches were mostly supplanted by supervised learning approaches [29–31]. Joint coreference resolution of all mentions in a document has also been explored [3]. Somewhat surprisingly, in recent years, systems based on deterministic rules have been shown to provide superior performance in coreference resolution tasks. Haghighi and Klein [32] proposed an algorithm that modularized syntactic, semantic, and discourse constraints on coreference and learned syntactic and semantic compatibility rules from a large, unlabeled corpus. Syntactic constraints included recognizing structures such as appositive constructions and i-within-i filter, which prevents parent noun phrases in a parse tree from being coreferent with any of children. Semantic compatibility of headwords and proper names were mined from Wikipedia and newswire texts. In a similar vein, Lee et al. [20] combine global information with simple but precise features in a *sieve* architecture. In the first stage of the architecture, mention detection is performed with high recall. The second stage consists of using precision-ranked coreference sieves (essentially deterministic models for exact string match, proper head noun match, etc.) and global information through an entity-centric clustering model. They achieved the best performance in the CoNLL 2011 shared task on unrestricted coreference resolution [13], a task that was based on the coreference annotations in the OntoNotes corpus [8]. Context-dependent semantics/discourse, coordinate noun phrases, and enumerations are given as the shortcomings of the system. The sieve architecture has been adopted by some of the most successful systems in the CoNLL 2012 shared task on multilingual coreference resolution, as well [2], and it has been made part of the Stanford CoreNLP toolkit [33]. In addition to these studies that focus on end-to-end coreference resolution, there is also a rich body of research on more specific aspects of coreference resolution, such as recognizing non-referential mentions (e.g., pleonastic *it*) [34], anaphoricity detection (i.e., determining whether a mention is anaphoric or not) [30, 35], distinguishing coreferential vs. singleton mentions [36], and resolution of discourse-deictic pronouns [37].

In the biomedical domain, early work focused on coreference resolution for biomedical literature. Castaño and Pustejovsky [15] described a rule-based system for resolution of pronominal and sortal (nominal) anaphora involving bio-entities (e.g., amino acids, proteins, cells). Their system makes heavy use of semantic type information from UMLS [38]. A resolution context is defined for each anaphor (e.g., the context for antecedents of reflexive pronouns is taken to be the current sentence) and all antecedent candidates in the context are scored based on their compatibility (e.g., number, person agreement, semantic preference) with the anaphor. The candidate with the highest cumulative salience score above a threshold is taken to be the antecedent. They annotated a corpus of 46 MEDLINE citations with anaphora information, achieving 73.8% F_1_ score. Kim et al. [16] augmented a system that extracts general biological interaction information with pronominal and nominal anaphora. For pronominal anaphora, they adopted Centering Theory and exploited syntactic parallelism of the anaphor and its antecedent. Their nominal anaphora resolution is similar to Castaño and Pustejovsky’s [15] salience measure-based approach; however, the former pay special attention to certain syntactic structures, such as coordinate noun phrases and appositive constructions. They reported precision of 75% and recall of 54% on a small MEDLINE dataset, yielding an F_1_ score of 63%. In contrast to these studies, Gasperin and Briscoe [39] focused on coreference in full-text biological articles and provided an annotated corpus of five articles on molecular biology of *Drosophila melanogaster*. They excluded pronominal anaphora relations from the annotation, due to their sparsity in biomedical literature, a fact confirmed by other studies, as well [40]. *Drosophila melanogaster* corpus is also unique in that three types of *associative* coreference are annotated: homology, related biotype (e.g., a gene and its product, a protein), and the set-membership relations. The first two can be considered specific to the biological domain. Their resolution approach uses a Bayesian probabilistic model and achieves an F_1_ score of 57% for coreference relations, but it is less successful in identifying biotype and set-membership relations. In the BioNLP 2011 shared task on event extraction [18], a supporting task on protein coreference resolution was proposed, based on the observation that one of major difficulties in event extraction is coreference resolution. A set of MEDLINE abstracts on transcription factors from the GENIA corpus [41] was annotated for protein coreference; pronominal anaphora as well as nominal anaphora were considered. Six systems participated in the task and the best system, an adaptation of a coreference resolution system developed for newswire text, achieved an F_1_ score of 34.05% [42], indicating a significant degradation in performance by the change of the domain. A similar trend was noted recently by Choi et al. [22], who used the state-of-the-art Stanford CoreNLP deterministic coreference resolution system [20] on this dataset and reported extremely poor results, partly attributed to lack of semantic knowledge usage. Nguyen et al. [17] achieved an F_1_ score of 62.4% on the same dataset, integrating domain-specific semantic information, confirming the more prominent role of semantic knowledge in biomedical coreference. Coreference information has also been integrated into event/relation extraction pipelines and varying degrees of improvements due to coreference resolution have been reported [43–46].

Coreference annotation in biomedical literature has mostly focused on annotating the mention pairs that corefer, rather than mention chains, which is the norm in the general domain. This is mostly driven by pragmatic concerns: often, coreference resolution is conceived as a task that supports other, more salient tasks, such as event extraction. In such cases, it is less important to identify the full mention cluster accurately than to identify one of the referents or, in the case of set-membership coreference, the member referents belonging to the set. Once the referents are identified, due to the transitive closure of the pairwise decisions, the mentions can mostly be substituted with the referents and the more salient tasks can be tackled in the usual manner. In addition, tasks that depend on coreference resolution often involve semantic components and will perform concept recognition/normalization, using tools such as MetaMap [47]. By addressing synonymy, such tools can aid in forming mention clusters, if needed. One exception to this pragmatic approach in studies focusing on literature is the coreference annotation in the CRAFT corpus [48], in which all mentions are annotated to form coreference chains in full-text articles. In addition, in contrast to other corpora, all semantic types are considered. CRAFT annotation guidelines mostly follow those of the OntoNotes corpus, although there are differences, such as CRAFT’s annotation of generics (such as bare plurals) as mentions. We are not aware of any coreference resolution study using this corpus. Another annotation effort (HANAPIN) focused on the biochemistry literature [49]. This corpus consists of 20 full-text, open-access articles. In addition to nominal and pronominal anaphora generally taken into account, abbreviation/acronyms as well as numerical coreference were annotated. A wider range of entity types, including drug effects and diseases, were considered.

The research in coreference resolution for clinical narratives began in earnest after the availability of the coreference annotation in the i2b2/VA corpus [19] and the ODIE corpus [50], both of which were used for training and evaluation in the 2011 i2b2/VA shared task [19]. Coreference annotation in these corpora is similar to that in the OntoNotes corpus, in that mention clusters are annotated. Entity types annotated include problem, person, test, treatment, anatomical site. Supervised learning, rule-based, and hybrid approaches were explored by the systems participating in the shared task; the best performance on the i2b2/VA corpus was reported by a supervised learning method incorporating document structure and world knowledge [51]. On the other hand, a rule-based system performed best on the ODIE corpus [52], while hybrid approaches using sieve architecture performed successfully overall [53, 54]. Attempts to use the existing general domain coreference tools for the task yielded poor results [21].

Coreference resolution has also been attempted in the so-called *gray literature* genre. For example, Segura-Bedmar et al. [55] reported a corpus of 49 drug interaction documents annotated with pronominal and nominal anaphora relations (DrugNerAR). Their resolution approach exploits Centering Theory for pronominal anaphora. For nominal anaphora, semantic information extracted from UMLS as well as information about drug classes are used. They report an F_1_ score of 0.76, a significant improvement over the baseline method (0.44), which consists of mapping the anaphors to the closest nominal phrases. Kilicoglu and Demner-Fushman [24] reported experiments on the DrugNerAR corpus as well as another similar corpus extracted from the DailyMed structured drug labels. Their results illustrated the importance of appositive constructions and coordinate noun phrases in resolving anaphora in drug-related texts. In another study, Kilicoglu et al. [23] investigated the role of anaphora and ellipsis in understanding consumer health questions and reported an 18 point improvement in F_1_ score in question frame extraction due to resolving pronominal and nominal anaphora.

## Materials and Methods

In this section, we first describe in detail our fine-grained coreference annotation of structured drug labels from DailyMed. Next, we discuss the smorgasbord architecture and individual methods currently used by resolution strategies, providing examples from this annotated dataset. We end the section with a brief discussion of evaluation metrics.

### Coreference Annotation of Structured Drug Labels

We annotated a set of structured drug labels (SPLs) extracted from DailyMed with several types of coreference relations. The annotated corpus consists of 181 labels for drugs that are used in the treatment of cardiovascular disorders, containing 348,513 tokens (approximately 1,925 tokens per label). This set was initially extracted from DailyMed for drug-drug interaction (DDI) extraction studies and was annotated with several entity and relation types [56]. In this earlier annotation study, the annotated entity types were drug, drug_class, and substance. Over the course of that annotation study, it was observed that coreference resolution would assist in the DDI extraction task and, therefore, some coreference relations were also annotated. However, the annotators were instructed to annotate coreference only when its resolution would be beneficial to the DDI task; therefore, coreference annotation in this corpus is sparse. For example, no coreference was annotated for the sentence below, even though there is a clear anaphoric relation between *the product* and its antecedent *Plavix*.

(3) [Plavix]_Antecedent_ is contraindicated in patients with hypersensitivity (e.g., anaphylaxis) to clopidogrel or any component of [the product]_Anaphor_

In the current work, we annotated coreference in this corpus regardless of whether it was useful for the DDI task or not, so that the dataset could be used to train and evaluate coreference resolution systems focusing on drug-related information. To make the task feasible while at the same time ensuring that the annotations could still be useful for learning algorithms, we limited coreference annotations to those that involve drugs and substances, ignoring coreference of other semantic types, such as disorders and procedures. While we constrained ourselves to this specific semantic group, we aimed at being general with respect to coreference types that could be annotated; we not only considered anaphora relations that are typically annotated in such corpora but also cataphora, appositive and predicate nominative relations, thus, taking into account both identity and appositive relations. Cataphora refers to coreference relations in which the referent (*consequent*) appears in the context following the coreferential mention (*cataphor*). We also annotated set-membership coreference, very prevalent in SPLs and resolution of which is critical for downstream tasks. Furthermore, we did not ignore generics (e.g., bare plurals), as was done in OntoNotes coreference annotation [2], agreeing with the rationale presented by Cohen et al. [48] with respect to lack of generics in biomedical text. On the other hand, indirect coreference relations [57], such as part/whole relations (meronymy) and bridging inference, and abbreviations/acronyms were left out of the scope of this project.

To facilitate the annotation task, we leveraged the entity annotations already present in the DDI corpus. In a pre-annotation step, we extracted all annotated entity strings in the corpus and, ignoring case differences, automatically annotated all unannotated occurrences of these strings with the entity type most frequently associated with it. For example, all mentions of *ACE inhibitors* were annotated as drug_class. This allowed the annotators to better focus on the coreference annotation task. The annotators were instructed to annotate entity mentions in case they were missed by this pre-annotation procedure. They were also instructed to correct/delete coreference annotations present from the previous study when necessary. Several documents were removed from the collection, since they overlapped significantly with other drug label documents. In pre-annotation, we also automatically removed coreference-related annotations (mention and coreference) that did not involve drugs or substances. For example, we deleted annotations associated with mentions such as *this process*, *such an effect*, which resulted in a reduction in the number of initial coreference-related annotations, in contrast to an increase in the number of entity-related annotations, as seen in Table 1:

**Table 1:** Number of annotations before and after pre-annotation.

Annotation Type	Original	With pre-annotations
drug	4621	13144
drug_class	2808	5635
substance	153	589
mention	262	198
coreference	431	352

In the current study, we view coreference annotation as a pairwise relation annotation task, rather than a coreference chain annotation task. This formulation is similar to that adopted in most biomedical corpora (e.g., [15, 18, 39]), and differs from most coreference corpora in the general domain (e.g., [8]). Like others [39], we take the view that from pairwise annotations, coreference chains can be largely inferred by merging the links between mentions of the same entity, with the aid of a concept normalization tool such as MetaMap [47]. This formulation also simplifies the annotation task significantly, since all the annotator needs to do is to link a mention to its closest referent.

The annotation was carried out by two annotators, a medical librarian and a physician with a degree in biomedical informatics. Both had been involved with the previous DDI annotation study, as well, and were familiar with the topics covered in the corpus. *brat* annotation tool [58] was used for annotation. The entire corpus was double-annotated and the annotations were later discussed and reconciled by both annotators. Disagreements were resolved by the authors of this paper. The annotation was completed in approximately 3 months, annotators splitting their time between this task and their other work-related activities. The basic instructions provided to the annotators are given in Appendix.

The annotation study proceeded in several steps:

1. **Practice phase**: Annotators were presented with basic annotation guidelines that provided definitions of the relevant phenomena and examples. Each annotator then annotated five drug label documents. Annotation differences were identified and the primary author (HK) and the annotators discussed these differences to further refine and clarify the guidelines.
2. **Main annotation phase**: Annotators annotated in batches of approximately 30 labels at a time. The annotators could discuss complex cases or ask for clarification. Once the annotation of a batch was completed by both annotators, inter-annotator agreement was calculated to assess the progress. The annotators then reconciled their annotations, consulting with the authors in the cases of disagreement. The guidelines were updated, if necessary.
3. **Semi-automated fine-grained annotation phase**: The primary author conducted this phase. All mention and coreference annotations were semi-automatically subcategorized into fine-grained classes, and each element of the coreference relations were labeled with the appropriate role (antecedent, cataphor, attribute, etc.). The motivation for this step is that it is generally accepted that different approaches are needed to detect and resolve different types of coreference (e.g., anaphora vs. appositive) indicated by different means (pronouns vs. noun phrases). These fine-grained annotations, then, may serve to train/evaluate such type-specific approaches. We also performed quality control of the annotations in this step. In this phase, the mention annotations were subcategorized into the following classes:
  - personal_pronoun (personal pronoun), possessive_pronoun, demonstrative_pronoun, relative_pronoun, indefinite_pronoun, distributive_pronoun, reciprocal_pronoun
  - definite_np(definite noun phrase), demonstrative_np, indefinite_np, distributive_np, zero_article_np
  The coreference annotations were subcategorized as follows, with the corresponding role labels given in parentheses. An example of each type is also given:
  - anaphora (Anaphor, Antecedent)
    - [Plavix]_Antecedent_ is contraindicated in patients with hypersensitivity (e.g., anaphylaxis) to clopidogrel or any component of [the product]_Anaphor_.
  - cataphora (Cataphor, Consequent)
    - Because of [its]_Cataphor_ relative beta1-selectivity, however, [Lopressor]_Consequent_ may be used with caution in patients with bronchospastic disease.
  - appositive (Attribute, Head)
    - the pharmacokinetics of [S-warfarin]_Head_ ([a CYP2C9 substrate]_Attribute_).
  - predicate_nominative (Attribute, Head)
    - [Clopidogrel]_Head_ is [a prodrug]_Attribute_.

The total number of resulting annotations in the corpus is given in Table 2. The fact that the number of coreference annotations is approximately one and a half times that of mention annotations is an indication of the prevalence of set-membership coreference in the corpus.

**Table 2:** Coarse-grained annotation counts.

Annotation Type	Number of annotations
drug	13124
drug_class	5628
substance	713
mention	1976
coreference	3006

The distribution of mention annotations after semi-automated fine-grained annotation is given in Table 3. The distribution shows the predominance of nominal mentions, even though personal and possessive pronouns also appear in more substantial quantities than they do in biomedical literature [39].

**Table 3:** Fine-grained coreferential mention counts.

Type	Number of annotations	%
personal_pronoun	196	9.9
possessive_pronoun	230	11.6
demonstrative_pronoun	8	0.4
relative_pronoun	32	1.6
indefinite_pronoun	3	0.2
distributive_pronoun	14	0.7
reciprocal_pronoun	6	0.3
definite_np	587	29.7
demonstrative_np	367	18.6
indefinite_np	328	16.6
distributive_np	61	3.1
zero_article_np	144	7.3

The distribution of coreference annotations after semi-automated fine-grained annotation is given in Table 4.

**Table 4:** After fine-grained annotation.

Coreference Type	Total	%	By Mention Type
anaphora	2021	67.2	definite NP (595), demonstrative NP (571), possessive pronoun (239), personal pronoun (205), distributive NP (131), zero article NP (116), indefinite NP (63), relative pronoun (49), distributive pronoun (28), reciprocal pronoun (12), demonstrative pronoun (9), indefinite pronoun (3)
cataphora	488	16.2	definite NP (419), demonstrative NP (34), indefinite NP (20), possessive pronoun (12), distributive NP (2), personal pronoun (1)
appositive	312	10.4	indefinite NP (146), zero article NP (106), definite NP (60)
predicate_nominative	185	6.2	indefinite NP (147), zero article NP (30), definite NP (8)

We calculated inter-annotator agreement after each batch of approximately 30 files was fully annotated. We take the F_1_ score when one set of annotations is taken as gold standard as the inter-annotator agreement, a measure often used in calculating relation annotation agreement in the absence of negative cases [59]. Agreement on both mentions and coreference relations was considered and both the exact match and the approximate match of the annotations were taken into account. In approximate matching, we considered two mentions to be a match, if their textual spans overlapped. Two coreference relations were considered to be a match, if both their mentions matched. Inter-annotator agreement calculations were based on coarse-grained annotations. The agreement results are given in Table 5. They show a clear improvement trend in inter-annotator agreement in the course of the study. The high agreement on the final batch is particularly encouraging, indicating that coreference can be reliably annotated by non-linguists provided with some training and guidelines.

**Table 5:** Inter-annotator agreement.

	Mention		Coreference	
Batch	Exact	Approximate	Exact	Approximate
1	0.6078	0.6976	0.4888	0.6485
2	0.7781	0.8138	0.6382	0.7083
3	0.7514	0.8171	0.5970	0.7164
4	0.8218	0.8764	0.7399	0.8074
5	0.8315	0.8853	0.7255	0.8309
6	0.9485	0.9708	0.8651	0.8921

### Bio-SCoRes Framework

**Fig 1:**
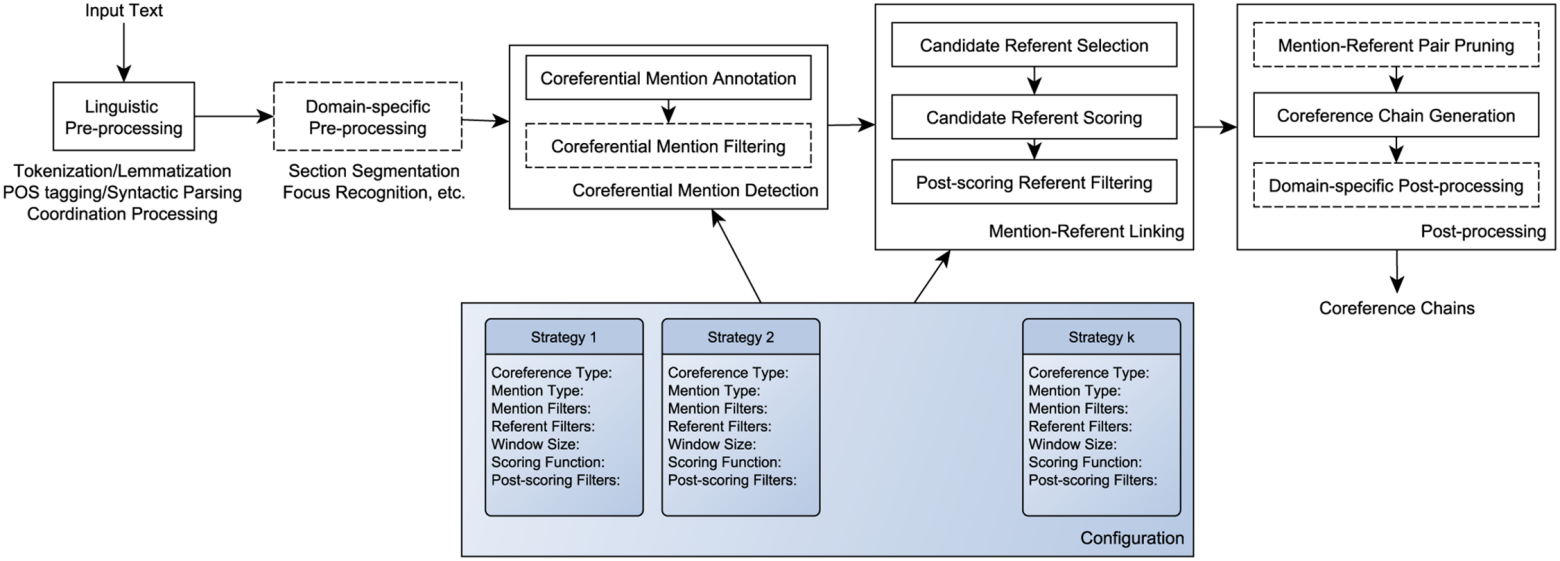
The high-level view of the Bio-SCoRes framework.

Our coreference resolution framework follows a pipeline architecture, consisting of several mandatory and optional steps. These steps are illustrated in Fig 1. The mandatory steps are illustrated as solid boxes, while the optional steps are shown as dotted boxes. The methodology is driven by *resolution strategies*, which collectively form a *configuration*. Each resolution strategy addresses coreference resolution of a specific mention type and declares a combination of methods that address a particular aspect of the coreference resolution process. These strategies are intended to be modular, so that new methods can be defined and they can be mixed and matched for different text types and domains in a smorgasbord style. In this subsection, in addition to discussing the components of the framework, we also describe the particular methods that we implemented to address coreference in SPLs, some of which were used for other text types, as well. In later sections, we will also describe the additions/modifications we made to this core pipeline to address coreference in biomedical literature and discharge summaries.

As can be seen from Fig 1, the mandatory components of the framework are linguistic pre-processing, coreferential mention detection, mention-referent linking and, largely corpus-specific, post-processing. Mention detection and mention-referent linking are at the core of the framework. On the other hand, optional components can be used for specific tasks that can aid in resolution, such as section segmentation, or in post-processing to prune unwanted coreference relations.

#### Linguistic Pre-processing

The framework presupposes linguistic processing of the input documents. It requires lemma, part-of-speech and character offsets for individual tokens as well offsets, syntactic parse trees and dependency relations for sentences. For the experiments reported in this paper, for this type of lexical and syntactic analysis, we used Stanford CoreNLP toolkit [33]. The framework does not perform named entity recognition; however, it is designed to accept named entity annotations extracted by an external system or assigned manually. Named entities are expected to have semantic type information (e.g., drug, substance, etc.). The system can also use an additional, coarser level of semantic typing called semantic groups, each of which can contain multiple more fine-grained semantic types. Even though it is possible to create a coreference resolution pipeline that does not use semantic information at all, this is likely to result in impoverished performance.

Once the lexical, syntactic, and semantic information for a document is obtained, we perform a series of syntactic dependency transformations, a subset of those specified in the Embedding Framework [60]. These syntactic transformations serve several purposes, such as a) integrating semantic information with the syntactic structure, b) addressing syntactic phenomena such as coordination, and c) correcting potential dependency parsing errors. We sequentially apply the following transformations in our pipeline:

- *Semantic Enrichment* allows merging named entity annotations with the tokens and into the dependency graph and results in a simplified dependency graph in which intra-entity dependencies are deleted.
- *PP-Attachment Correction* attempts to correct prepositional phrase attachment errors, generated frequently by syntactic parsers, using deterministic rules discussed in Schuman and Bergler [61].
- *Modifier Coordination Transformation* rearranges dependency relations involving named entities that appear in pre-modifier position of noun phrases. This is mostly a corrective transformation.
- *Term Coordination Transformation* attempts to address complex, serial coordination cases involving named entities, by exploiting information about punctuations and parentheticals.
- *Coordination Transformation* identifies the arguments of a conjunction and modifies the dependency graph to better reflect the semantic dependencies between the conjunction and its arguments.
- *NP-Internal Transformation* performs a kind of NP-chunking using dependency relations and semantic information, such as named entities.

Using the transformed dependencies from this step, we next recognize and explicitly represent the coordinated named entities. In addition to being syntactically coordinated, we also require that the named entities be semantically compatible. Syntactic coordination information is directly derived from the modified dependency graph. Two syntactically coordinated named entities are taken to be compatible, if they share a semantic type/group. These coordination-specific steps are expected to aid in resolution of set-membership coreference, a common occurrence in biomedical text.

#### Domain-specific Pre-processing

While certain types of pre-processing can be useful for coreference resolution in general (such as coordination processing), others can aid the task in specific text types or domains. We refer to such optional steps that can aid the resolution process as *domain-specific pre-processing*. For example, it is widely accepted that discourse structure and coreference are closely linked; in other words, discourse structure may constrain where the referents of a mention can be found [62]. For SPLs and clinical reports, which can be lengthy and are generally composed of multiple sections, it can be useful to segment the text into sections to constrain the search space. Another example of a domain-specific pre-processing is recognition of the document topic (focus). For example, drug labels describe a single drug and its ingredients, and it could help the resolution process to recognize these terms.

For our experiments on SPLs, we performed section segmentation. To accomplish this, we simply compiled a list of section headers used in the labels, including DRUG INTERACTIONS, PRECAUTIONS, and WARNINGS, and used simple regular expressions to recognize them.

Another pre-processing step involved recognition of discourse-level coordination and was performed mainly to improve cataphora resolution. An example of discourse-level coordination is given below.

(4) When administered concurrently, [the following drugs]_Cataphor_ may interact with thiazide diuretics.
  - [Antidiabetic drugs (oral agents]_Consequent_ and [insulin]_Consequent_): Dosage adjustment of the antidiabetic drug may be required.
  - [Lithium]_Consequent_: Diuretic agents increase the risk of lithium toxicity. …
  - [Nonsteroidal anti-inflammatory drugs]_Consequent_ (NSAIDs) and [COX-2 selective agents]_Consequent_: When Amturnide and nonsteroidal anti-inflammatory agents are used concomitantly, …

To resolve such instances, we need to recognize that the consequents are part of an itemized list and they should be treated as conjuncts. The list recognition step presupposes that the label is segmented into sections. We group all the terms in a given section by their semantic group. Iterating through each term group, we check whether a term in the group is the first element on its line and, if so, add it to the discourse-level conjunct list, with respect to its semantic group. If the term itself is coordinated with others on the same line, they are added to the conjunct list, as well.

#### Configuring Resolution Strategies

The core coreference resolution steps, mention detection and mention-referent linking, are driven by a *configuration* determined according to specific needs of the corpus or task, that consists of a set of *resolution strategies*. Taken together, resolution strategies determine the coreference types to be addressed by the framework and the mention types to consider for each coreference type. For example, our coreference resolution pipeline for SPLs recognizes the following mention types: pronouns (personal, possessive, distributive, and reciprocal) and noun phrases (definite, demonstrative, distributive, indefinite, and zero article). Though annotated, demonstrative pronouns and indefinite pronouns are not considered, since they are rarely used to indicate drug coreference. The pipeline addresses four types of coreference relations annotated in the SPLs: anaphora, cataphora, appositive, and predicate nominative.

Resolution strategies are declarative and each consists of the following elements:

- *Coreference type* indicates the type of coreference relation the strategy addresses (e.g., anaphora, cataphora).
- *Mention type* indicates the syntactic class of the mentions the strategy addresses (e.g., personal pronoun, definite noun phrase).
- *Coreferential mention filters* indicate the methods to eliminate unwanted mention annotations from coreference consideration (e.g., elimination of *rigid designators* [15], such as *the p38 MAP kinase*).
- *Candidate referent filters* indicate the filtering methods to eliminate candidate referents incompatible with the mention on some constraint (e.g., elimination of candidates that occur after the mention for anaphora cases, elimination of candidates outside a predefined window).
- *Scoring function* is used to calculate a cumulative score for the compatibility between a coreferential mention and a candidate referent. Compatibility is measured using a selection of *agreement methods*, each associated with a reward for agreement and a penalty for disagreement. Given a mention *m* and a candidate referent *c*, the compatibility score of *c* with respect to the mention *m* (*SC(m,c)*) using a set of *k* agreement methods *AM*_1…k_(*M,C*) can be given recursively as:
  $$SC_{k}\left(c,m\right)=SC_{k-1}\left(c,m\right)+\{\begin{array}{c} Rew\left({AM}_{k}\right),\text{if}AM_{k}\left(c,m\right)=1\\ -Pen({AM}_{k}),\text{otherwise} \end{array}$$
  where *Rew*(*AM*_*k*_) indicates the reward for agreement on *AM*, and *Pen*(*AM*_*k*_) the penalty for disagreement. Initially,
  $$SC_{0}\left(c,m\right)=0$$
  Scoring function is inspired by the salience measures used by Lappin and Leass [27] as well as Castaño and Pustejovsky [15]. Reward and penalty values can be used to encode hard and soft constraints.
- *Post-scoring referent filters* indicate the methods to use to identify the best referent among a set of scored candidate referents. Often, a filtering method based on scores is combined with another salience measure to break ties (e.g., a threshold-based filter followed by a proximity filter).

As an example, the strategy used in anaphora resolution of personal pronouns in SPLs is given in Table 6. This strategy indicates the following:

- It is applicable for anaphora resolution of personal pronouns (Coreference Type and Mention Type).
- Only third person personal pronouns (e.g., *it, they, them*) are considered and pleonastic *it* pronouns (as in *it is accepted that …*) are ignored (Mention Filters).
- Candidate antecedent selection is based on running five filters sequentially over the input text (Candidate Filters). For instance, one of these, PriorDiscourse filter, will remove the candidates that do not precede the anaphor and another, WindowSize(2), will remove candidates that are not within two sentences of the mention.
- The antecedent scoring is based on its compatibility with the anaphor on four agreement constraints (Scoring Function), including number agreement. If the antecedent and the anaphor agree on number (either both are plural or both are singular), the score is incremented by 1. No penalty is incurred, when the antecedent and the anaphor do not agree on number (0).
- After the candidate referents are scored, those with a score less than 4 are eliminated (Threshold(4)) and highest-ranked candidates are kept. If there are multiple candidates at this point, the one closest to the anaphor mention over the parse tree (Salience(Parse)) is selected as the best candidate. If no candidates have scores over 4, no coreference link is generated.

**Table 6:** Resolution strategy for personal pronominal anaphora.

*Coreference Type*	Anaphora
*Mention Type*	PersonalPronoun
*Mention Filters*	ThirdPerson, PleonasticIt
*Candidate Referent Filters*	PriorDiscourse, WindowSize(2), SyntacticConfig, Default, Exemplification
*Scoring Function*	Person(1,0) + Gender(1,0) + Animacy(1,0) + Number(1,0)
*Post-scoring Filters*	Threshold(4), TopScore, Salience(Parse)

In the scope of this study, we defined a number of methods for mention filtering, candidate referent filtering, agreement, and post-scoring filtering to use in coreference pipelines. The framework allows defining new filters and constraints, as well. We will describe these methods, when appropriate, in the following sections. The full configuration for coreference resolution of SPLs is given in Table 7.

**Table 7:** Configuration for structured drug label coreference resolution.

Mention Type	Mention Filters	Referent Filters	Agreement Methods	Post-Scoring Filters
*Anaphora resolution*				
PersonalPronoun	ThirdPerson	PriorDiscourse	Person(1,0)	Threshold(4)
	PleonasticIt	WindowSize(2)	Gender(1,0)	TopScore
		SyntacticConfig	Animacy(1,0)	Salience(Parse)
		Default	Number(1,0)	
		Exemplification		
PossessivePronoun	ThirdPerson	PriorDiscourse	Person(1,0)	Threshold(4)
		WindowSize(2)	Gender(1,0)	TopScore
		Default	Animacy(1,0)	Salience(Parse)
		Exemplification	Number(1,0)	
DistributivePronoun	None	PriorDiscourse	Person(1,0)	Threshold(4)
ReciprocalPronoun		WindowSize(2)	Gender(1,0)	TopScore
		SyntacticConfig	Animacy(1,0)	Salience(Default)
		Default	Number(1,0)	
		Exemplification		
DefiniteNP	Anaphoricity	PriorDiscourse	Number(1,1)	Threshold(4)
DemonstrativeNP		SyntacticConfig	HypernymList(3,0)	TopScore
DistributiveNP		Default		Salience(Default)
		Exemplification		
*Cataphora resolution*				
PersonalPronoun	ThirdPerson	SubsequentDiscourse	Person(1,0)	Threshold(4)
	PleonasticIt	WindowSize(Sentence)	Gender(1,0)	TopScore
		SyntacticConfig	Animacy(1,0)	Salience(Default)
		Default	Number(1,0)	
PossessivePronoun	ThirdPerson	SubsequentDiscourse	Person(1,0)	Threshold(4)
		WindowSize(Sentence)	Gender(1,0)	TopScore
		Default	Animacy(1,0)	Salience(Default)
			Number(1,0)	
			DiscourseConnective(1,2)	
DefiniteNP	Cataphoricity	SubsequentDiscourse	Number(1,1)	Threshold(4)
		SyntacticConfig	HypernymList(3,0)	TopScore
		WindowSize(2)		Salience(Default)
		Default		
*Appositive resolution*				
DefiniteNP	None	WindowSize(Sentence)	Number(1,1)	Threshold(4)
IndefiniteNP		Default	SyntacticAppositive(3,2)	TopScore
ZeroArticleNP			HypernymList(1,1)	Salience(Default)
*Predicate nominative resolution*				
IndefiniteNP	None	PriorDiscourse	Number(1,1)	Threshold(4)
ZeroArticleNP		WindowSize(Sentence)	SyntacticPredicate	TopScore
		Default	Nominative(3,2)	Salience(Default)
			HypernymList(1,1)	

#### Coreferential Mention Detection

The first phase of coreference resolution is concerned with annotating and filtering the coreferential mentions included in the resolution strategies (illustrated as Coreferential Mention Annotation and Coreferential Mention Filtering in Fig 1). The annotation is rule-based and relies on lexical and syntactic cues. We examine all tokens in text and annotate them with the appropriate mention type, if they satisfy the appropriate constraints, as described below:

- *Personal and possessive pronouns:* Personal and possessive pronouns are detected based simply on their part-of-speech (PRP and PRP$, respectively). Reflexive pronouns are also marked as such, based on their token (*itself, themselves*).
- *Demonstrative pronouns:* Demonstrative pronouns (*this, that, these, those*) are recognized based on their tokens. We further require that the demonstrative pronoun *that* is not used as a complementizer (as in *The results show that …*) and that these pronouns are not in the determiner position of a noun phrase. These rules are implemented using dependency relations.
- *Distributive pronouns:* Distributive pronouns (*both, either, neither, each*) are also determined based on the dependency relations they occur in. We require that the pronoun under consideration is neither in the dependent position in a *preconj* (preconjunct) dependency nor in the head position of a *pobj* (object of preposition) relation, to exclude phrases like *both INDOCIN I.V. and furosemide* from consideration.
- *Reciprocal pronouns:* We determine reciprocal pronouns (*each other, one another*) based on simple string match, ignoring case.
- *Indefinite pronouns:* Indefinite pronouns are also determined based on their tokens (*another, some, all*) and their part-of-speech (DT).
- *Relative pronouns:* Relative pronouns (e.g., *who, which, whose, that*) are recognized based on their part-of-speech (WDT, WRB, WP, or WP$, and *IN* for *that*). We further require that *that* is not used as a complementizer.
- *Definite noun phrases:* One of the syntactic transformations (NP-Internal Transformation) chunks noun phrases in a sentence. We mark as definite noun phrases those that begin with the definite article, *the*.
- *Demonstrative noun phrases:* Similarly to definite noun phrases, we mark as demonstrative noun phrases those that begin with the demonstrative determiners, *this, that, these, those*, or with the demonstrative adjective, *such*.
- *Distributive noun phrases:* We mark those that begin with the distributive determiners *either, neither, both, each*.
- *Indefinite noun phrases:* We mark those that begin with the indefinite articles *a, an*, indefinite determiners *any, some, all, another*, and with the indefinite adjective, *other*.
- *Zero article noun phrases:* These are distinguished by the absence of any triggers for other noun phrase types. For precision, we also require that the head of the noun phrase be a hypernym for the semantic type/group it belongs to (e.g., *medication* is a hypernym for the drug semantic type). The system currently provides a small list of such hypernyms for several semantic groups, which can be extended/redefined.

After mention types are annotated, mention filters are applied to eliminate from further consideration mentions that are not coreferential or not in the scope of resolution. Four mention filters that were used in SPLs were ThirdPronoun, PleonasticIt, Anaphoricity, and Cataphoricity filters. The first filter (ThirdPronoun) applies to personal and possessive pronouns and eliminates first and second person pronouns (such as *I*, *me*, *you*, *yourself*) from consideration, since the corpus is concerned with drug/substance coreference only. The second (PleonasticIt) applies to personal pronouns only and removes *it* mentions that are pleonastic, and therefore, not coreferential. An example of pleonastic *it* is given below.

(5) It is not certain that these events were attributable to DEMADEX.

Pleonastic *it* is recognized using a simple dependency-based rule that mimics patterns in Segura-Bedmar et al. [55], given below.

nsubj*(X,it) ∧ DEP(X,Y) ⇒ PLEONASTIC(it)

where nsubj* refers to *nsubj* or *nsubjpass* dependencies and *DEP* is any dependency, where *DEP* ∉ {*infmod*, *ccomp*, *xcomp*, *vmod*}.

Anaphoricity and Cataphoricity mention filters are largely similar. They both eliminate noun phrases that correspond to *rigid designators* [15] and noun phrases that are in appositive constructions. For example, the definite noun phrase *the nitric oxide/cGMP pathway* is eliminated since it is a rigid designator, while *the monotherapies* in the following sentence is not considered for anaphora, since it is in an appositive construction with the coordinate noun phrase *aliskiren and valsartan*.

(6) the incidence of hyperkalemia …was about 1%-2% higher in the combination treatment group compared with the monotherapies aliskiren and valsartan, or with placebo

In addition to these two constraints, Anaphoricity filter eliminates noun phrases with the word *following* (e.g., *the following drugs*) and the noun phrase that begins a document, since they can only be cataphoric. Cataphoricity filter, on the other hand, keeps those noun phrases eliminated by this constraint, and discards the rest.

#### Mention-Referent Linking

After the relevant coreferential mentions are selected, we attempt to link them to their referents. The system proceeds from left to right and as coreferential mentions are encountered, it attempts to resolve their referents based on their mention types, the kinds of coreference the system aims to resolve, and the parameters of the appropriate resolution strategies. This phase consists of the following steps: Candidate Referent Selection, Candidate Referent Scoring, and Post-scoring Referent Filtering.

Candidate Referent Selection step is concerned with identifying all the candidate referents for a given mention, and is driven by *candidate referent filters* parameter of the strategy being applied. Filters are applied sequentially. The following referent filters are used for selection in structured drug labels:

- PriorDiscourse: This filter includes as candidates those mentions that precede the coreferential mention. It is applicable for anaphora resolution.
- WindowSize: This filter includes as candidates those mentions that are within a predefined number of sentences from the mention. For example, if the window size parameter is 2, candidates are taken to be the mentions that are within two sentences from the coreferential mention. If not used, all document text is used for candidate selection. Sentence and Section can also be specified as the window.
- SyntacticConfiguration: This filter removes candidates that are in a particular syntactic configuration with the coreferential mention that would render them impossible to corefer. This filter is generally not applicable to appositive and predicate nominative types as well as to possessive and relative pronouns, since in these cases, the candidates may be linked to the mention via syntactic means. This filter works by identifying the syntactic dependency path between the coreferential mention and the candidate referent. If a path is not found, the candidate is included. If a path is found, the candidate is excluded if:
  - There is verbal path and the candidate and the mention are in the subject and object positions of a verb.
  - There is a nominal path and the candidate and the mention are in the subject and object position of a nominal predicate.
  - If the length of the path is at most 2 and one of the dependencies indicates an appositive construction.
  - If the length of the path is at most 2 and ignoring those indicating an appositive construction, all dependencies indicate subject, object, prepositional phrase, or coordination constructions.
  This filter is a somewhat modified version of i-within-i filter used in [32], among others.
- NounPhrase: This filter includes only noun phrases, identified in linguistic pre-processing, as candidate referents.
- VerbPhrase: This filter includes heads of verb phrases as candidates.
- SemanticClass: This filter includes semantic objects of specific, predefined classes as candidates (e.g., named entities, events).
- Default: This filter combines the previous three filters to include candidate referents that pass NounPhrase and SemanticClass filters and exclude those that pass VerbPhrase filter. For SPLs, SemanticClass filter allows named entities, coreferential mentions, and conjunctions indicating a collection of named entities.
- Exemplification: This filter is concerned with set-membership relations and removes from consideration candidates that are specific instantiations of a larger class, also mentioned in text. In the following example, the drugs and drug classes in parentheses are not considered as candidates for the personal pronoun *they*, whereas the class that they belong to (i.e., *Inhibitors of this isoenzyme*) is.
  (7) [Inhibitors of this isoenzyme]_Antecedent_ (e.g., macrolide antibiotics, azole antifungal agents, protease inhibitors, serotonin reuptake inhibitors, amiodarone, cannabinoids, diltiazem, grapefruit juice, nefazadone, norfloxacin, quinine, zafirlukast) should be cautiously co-administered with TIKOSYN as [they]_Anaphor_ can potentially increase dofetilide levels.
- SubsequentDiscourse: This filter is similar to PriorDiscourse filter, but it includes the candidates occurring subsequent to the mention, instead. This filter is appropriate for cataphora resolution.

Once a final candidate list is formed by applying the filters above, agreement methods are used to determine the level of compatibility between each candidate and the mention and the candidate is assigned a score. This step (Candidate Referent Scoring) is also driven by the appropriate resolution strategy. If a candidate and the mention are compatible according to a specific agreement method, then the candidate score is incremented by the reward value of the agreement method. Otherwise, the specified penalty is applied, if any, and the overall compatibility score may be lowered. In this section, we provide more details regarding the agreement methods used for SPLs.

- Number: This agreement indicates whether the candidate and the mention agree on their number. We calculate the number as either *plural* or *singular*. The number is taken as plural for conjunction candidates, for reciprocal and distributive pronouns, plural pronouns (e.g., *ours, they, etc.*), and collective nouns (e.g., *family, population, etc.*). Excluding these special cases, the part-of-speech tag is used to determine the number feature, if the mention is a noun. Otherwise, the number feature is taken as singular. For reciprocal and distributive pronouns or mentions with the word *two* (e.g., *these two enzymes*), we stipulate that the candidate be a coordinate noun phrase with two named entities, for agreement.
- Animacy: We calculate three animacy values: Animate, NotAnimate, and MaybeAnimate. For example, personal pronouns *he* and *she* and related forms are assigned the value Animate, while *it* and related forms are assigned NotAnimate. On the other hand, *they* and related forms are assigned the value MaybeAnimate. If a population semantic group is defined and the mention in consideration belongs to this semantic group, it is taken as Animate, as well.
- Gender: We calculate three gender values: Female, Male and Unknown. Personal pronoun *he* and related forms are assigned the value Male, whereas *she* and related forms are assigned Female and *it* and related forms are assigned Unknown. A small list of Female and Male words (such as *mother*, *father*, *son*) is also consulted. Gender agreement is predicted when the genders of the candidate and the mention match or they are both Unknown.
- Person: This agreement indicates whether the candidate and the mention agree on grammatical person, which takes the values First, Second, or Third. We determine the values First and Second by simple regular expressions and otherwise assign the value Third.
- HypernymList: This agreement is meaningful when the mention is nominal. It predicts that a candidate with a certain semantic type is in agreement with a mention if the headword of the mention is one of the explicitly defined hypernym words for that semantic type. For example, *clopidogrel* and *the medication* are compatible according to this agreement method, because *medication* is explicitly defined as a high-level term for the drug semantic type. Other hypernyms for this type include *drug, agent, compound, solution, product*, among others, and have been mined from the training data.
- DiscourseConnective: This agreement is concerned with cataphoric uses of personal and possessive pronouns, constrained with sentence-initial discourse connectives. It allows a personal/possessive pronoun to be cataphoric, if the sentence it appears in begins with one of the specified discourse connectives (*because*, *although*, *since*). In the following example, the discourse connective *because of* licenses the possessive pronoun *its* to be cataphoric.
  (8) *Because of [its]_Cataphor_ beta1-selectivity, this is less likely with [ZEBETA]_Consequent_*.
- SyntacticAppositive: This agreement is satisfied if the mention and the candidate are in a syntactic appositive construction. In the case the candidate referent is a conjunction, it is sufficient to have one of its conjuncts be in the appositive construction with the mention. Appositive constructions are determined by the presence of a dependency relation of type *appos* (appositive) or *abbrev* (abbreviation) between the mention and the referent. To increase recall and deal with often erroneous dependency relations, we also take contiguous noun phrases as appositives. For example, in the fragment *the ACE inhibitor enalapril*, the noun phrases *the ACE inhbitor* and *enalapril* are taken to be appositives.
- SyntacticPredicateNominative: This agreement is satisfied if the mention and the candidate are in a copular construction. To determine such constructions, we search for the dependency relation *cop* (copula) and ignore the cases in which object of the copular construction is negated (*neg* dependency). If a copular construction exists between the mention and the candidate, we stipulate that the candidate be in the subject position.

After each candidate is scored, the next step is selecting the best candidate as the referent using Post-scoring Referent Filtering. Similarly to previous steps, this is also driven by the appropriate resolution strategy, specifically, its *post-scoring filters* parameter. The methods specified in this parameter are applied sequentially over the candidate list. If no candidate is left after these filters are applied, no coreference link will be generated. The post-scoring filtering methods used in coreference resolution of SPLs are given below.

- Threshold: This filter discards all candidates with a score less than the given threshold.
- TopScore: This filter eliminates all candidates, except those with the highest score.
- Salience: This filter finds the candidates that are most salient to the mention on a specified measure and eliminates the rest. Default salience measure is proximity to the mention in number of intervening textual units, often used to break ties between compatible candidates. We also use Parse measure, which indicates the path distance from the mention to the referent on the syntactic parse tree. If the candidate and the mention are in different sentences, the sentence distance is added to the distance, as well, with a factor of 2. Using parse tree distance to calculate salience has been shown to be useful especially for pronominal anaphora [5, 26, 32, 55] and reflects the intuition of the Centering Theory [28]. If using the Parse measure results in a tie, we use the Default salience measure to select the best candidate.

#### Post-processing

Mention-Referent Linking phase results in a list of mention-best referent pairs. The framework does not make assumptions regarding how to generate coreference links from these pairs; however, in general, we expect that some kind of pruning may take place to eliminate pairs that are not of interest or are inconsistent (Mention-Referent Pruning), followed by generation of a new coreference chain or merging with an existing chains (Coreference Chain Generation) and other domain-specific post-processing. We describe these steps for SPLs below. Note that each coreference resolution pipeline based on the Bio-SCoRes framework is likely to have different mechanisms for this phase.

In Mention-Referent Pruning for SPLs, we address the personal and possessive pronouns that are involved in both anaphora and cataphora relations, due to DiscourseConnective agreement measure described earlier. In the example below, an anaphoric pair *{its,insulin}* and a cataphoric pair *{its,ZEBETA}* have been identified on the same pronoun.

(9) *Nonselective beta-blockers may potentiate insulin-induced hypoglycemia and delay recovery of serum glucose levels. Because of [its]_Cataphor_ beta1-selectivity, this is less likely with [ZEBETA]_Consequent_*.

In this step, we eliminate the anaphoric pair, since we find such pairs less likely to be correct than the cataphoric pairs in these instances.

In the SPL corpus, coreference is annotated as a pairwise binary relation between two terms (mention and referent). If the coreference is a set-membership coreference (i.e., a referent is a coordinate noun phrase with multiple named entity terms), a separate relation is annotated between the mention and each conjunct. In Coreference Chain Generation step, we split such referents into multiple mention-referent pairs, and simply generate a binary relation between the elements of the pair.

In the final domain-specific post-processing step, we prune the coreference relations that do not involve drugs/substances, since they were not annotated in the corpus. In the example below, the coreference relation shown is pruned even though it is accurate, because it is one that is not in the scope of the annotation.

(10) *Discuss with [patients]_Antecedent_ the appropriate action to take in the event that [they]_Anaphor_ experience anginal chest pain requiring nitroglycerin following intake of ADCIRCA*.

It is conceivable to perform pruning and some other aspects of domain-specific post-processing using various types of filters described earlier. For example, *patients* in the example above can be filtered out as a candidate using a candidate filtering method that relies on semantic type information. However, this has the drawback of potentially identifying another compatible mention, appearing before *patients* in text, as the referent. Therefore, it generally makes more sense to address coreference of all semantic types in mention-referent linking step, if possible, and perform pruning as a post-processing step. However, as we will show later, we have also used such filtering methods (based on semantic type/group) in other experiments, when appropriate. The smorgasbord architecture allows mixing and matching various filters for optimal results.

So far, we have described the framework as it was applied to coreference resolution in SPLs using gold entity mentions. We have performed various other experiments on this corpus, as well as experiments on two other coreference corpora, a corpus of i2b2/VA discharge summaries and a corpus of MEDLINE abstracts. Instead of describing the adaptation of the framework to these tasks in this section, we will discuss them in the Results section, when appropriate, for readability.

### Evaluation

We evaluated the results generated by our approach for structured drug labels using standard evaluation metrics, precision, recall, and F_1_ score. In this, we followed the evaluation method adopted for the BioNLP-ST 2011 task on coreference resolution [18], since our annotation methodology was similar to theirs. We evaluated our results on MEDLINE abstracts in the same way. To evaluate the results on the i2b2/VA discharge summaries, we used unweighted *F*_1_average over B-CUBED [10], MUC [9], and CEAF [11] metrics, adopted for the i2b2 challenge on coreference resolution [19], based on the same corpus. Briefly, B-CUBED evaluation measures the overlap between the coreference chains predicted and the gold standard chains, while MUC measures the minimum number of pair addition and removals required for them to match the gold chains. On the other hand, CEAF computes an optimal alignment between the predicted chains and the gold standard chains based on a similarity score.

## Results and Discussion

In this section, we first discuss the results we obtained on SPLs, using the methodology described in the previous section and analyze some of the errors that the system makes. Next, we describe the adaptation of this methodology to discharge summaries and biomedical literature and discuss the results for these adaptations.

### Coreference Resolution on Structured Drug Labels

We split the SPL corpus into training and test subsets and developed the resolution configuration discussed earlier and provided in Table 7, based on the training set. The training set consists of 109 labels and the test set of 72 labels. In the training set, 1358 of the coreference relations are anaphora instances, 312 are cataphora, 203 are appositive, and 113 are predicate nominatives. The corresponding number of coreference instances in the test set are 663, 176, 109, and 72, respectively.

In the first set of experiments, we presuppose that gold named entities (drug, drug_class, and substance types) are known. In another set of experiments, we presuppose that both gold named entities and gold coreferential mentions are known, to assess the mention-referent linking step only. Finally, we experiment with a named entity recognition/concept normalization system for end-to-end coreference resolution on this dataset.

The evaluation results concerning mention detection are shown in Table 8. No results are shown for demonstrative, relative, and indefinite pronouns, since the system did not attempt to detect them, and for reciprocal pronouns, since they did not appear in the test set.

**Table 8:** Evaluation results for mention detection on the test portion of SPL coreference dataset.

	Precision	Recall	F_1_
PersonalPronoun	96.8	100.0	98.4
PossessivePronoun	95.7	63.8	76.5
DistributivePronoun	33.3	100.0	50.0
DefiniteNP	85.0	94.6	89.6
DemonstrativeNP	93.8	96.0	94.9
DistributiveNP	81.3	59.1	68.4
IndefiniteNP	79.1	72.0	75.4
ZeroArticleNP	41.2	59.2	48.6
Overall	**80.1**	**82.3**	**81.2**

The results show that while we achieve good precision and recall for certain types of mentions (personal pronouns, for example), there is clearly room for improvement in detecting other types, particularly distributive pronouns/noun phrases and zero article noun phrases. Most of the errors made in mention detection step were found to be due to erroneous or unspecific dependency relations; for example, some distributive noun phrases were recognized as distributive pronouns, due to absence of required dependency relations, leading to low precision in detecting distributive pronouns and low recall in detecting distributive noun phrases. Ignoring demonstrative, relative, and indefinite pronouns, which we did not attempt to identify, the recall increases from 82.3 to 83.7 and the F_1_ score from 81.2 to 81.9.

As baseline for coreference resolution, we implemented a simple mechanism which, after noun phrase chunking, identifies coreferential mentions by the presence of certain determiners/pronouns and takes the closest mention to the left with a drug semantic type as the referent. This is roughly the same baseline that has been used in Segura-Bedmar et al. [55]. We also tried a baseline using proximity on either side to account for cataphoric/appositive instances, but this yields poorer results, since anaphora instances are much more prevalent.

Using the configuration given above, we obtain the results given in Table 9 on the test set using gold entity mentions. The number of instances for coreference/mention type pairs are given in parentheses. Evaluation is based on type and approximate span matching of the coreference elements.

**Table 9:** Evaluation results for coreference resolution on the test portion of SPL coreference dataset.

	Precision	Recall	F_1_
Baseline	6.0	35.6	10.3
Bio-SCoRes	**65.5**	**45.2**	**53.5**
- Anaphora	64.8	45.3	53.3
— PersonalPronoun (60)	82.9	48.3	61.1
— PossessivePronoun (74)	76.1	47.3	58.3
— DistributivePronoun (12)	45.4	83.3	58.8
— DefiniteNP (219)	55.1	44.3	49.1
— DemonstrativeNP (190)	74.4	62.6	68.0
— DistributiveNP (40)	41.7	25.0	31.3
- Cataphora	61.1	37.5	46.5
— PersonalPronoun (1)	100.0	100.0	100.0
— PossessivePronoun (6)	100.0	100.0	100.0
— DefiniteNP (135)	58.4	43.7	50.0
- Appositive	61.1	50.5	55.3
— DefiniteNP (24)	86.7	54.2	66.7
— IndefiniteNP (40)	91.7	55.0	68.8
— ZeroArticleNP (45)	39.2	44.4	41.7
- PredicateNominative	93.0	55.6	69.6
— IndefiniteNP (47)	96.8	63.9	76.9
— ZeroArticleNP (24)	83.3	41.7	55.6

The system performs significantly better than the baseline in this setting. As might be expected from a deterministic, rule-based approach, the system achieves better precision than recall. Predicate nominative instances are considerably easier to resolve, while cataphora and anaphora cases are more difficult. Even though appositive instances behave similarly to predicate nominatives to a large extent, the system does not perform as well on them. Some coreference/mention type combinations are especially challenging and/or rare, and we have not included them in the final configuration. For example, we have not attempted to resolve demonstrative pronouns, since they are often discourse-deictic [63] and rarely refer to drugs in the corpus. We also have not attempted to resolve indefinite pronouns, which are rarely coreferential in text, and relative pronouns, which were sparsely annotated in the corpus. We attempted to resolve indefinite and zero article noun phrases only in the context of appositives and predicate nominatives, since, in such cases, they are syntactically more constrained. Limiting the evaluation to only those coreference type/mention combinations that we in fact addressed, the overall recall increases from 45.2 to 50.3 and F_1_ from 53.5 to 56.9 (56.7 for anaphora, 52.8 for cataphora, and 70.2 for predicate nominative; recall and F_1_ score for appositive class remains the same). The largest increase is for the cataphora class, due to a single instance of cataphora that involved 34 consequent drug names linked to a single demonstrative noun phrase, illustrating also the importance coordination recognition for this task. In fact, turning coordination recognition off completely in pre-processing results in poor results, overall F_1_ score is reduced to 41.7, with the most dramatic effect on anaphora and cataphora classes (F_1_ score of 43.1 for anaphora and 13.8 for cataphora).

To assess mention-referent linking only, we evaluated the system using gold coreferential mentions, as well, and obtained the results in Table 10. In this setting, the baseline performs much better, indicating that simple keyword search for mentions (the previous baseline method) is inadequate and that coreference links can be recovered successfully, to some extent, without using sophisticated techniques if the coreferential mentions were identified accurately. The system also performs better with gold coreferential mentions; however, the improvement is not as significant as it is with the baseline. Precision is improved across all coreference types, while recall increases only for predicate nominative and anaphora types. The recall drop in appositive type seems to be due to the interaction of syntactic dependency transformation and coreferential mentions.

**Table 10:** Evaluation results on the test portion of SPL coreference dataset using gold coreferential mentions.

	Precision	Recall	F_1_
Baseline	66.7	38.6	48.9
Bio-SCoRes	**77.3**	**48.6**	**59.7**
- Anaphora	76.9	50.7	61.1
- Cataphora	62.3	37.5	46.8
- Appositive	91.9	41.3	57.0
- PredicateNominative	98.0	68.1	80.3

We also evaluated the performance of an end-to-end coreference resolution pipeline. To do this, we augmented the pipeline with the MetaMap tool [47] to map drug label text to UMLS Metathesaurus concepts [38] and ran two experiments. In the first, we used the configuration that yielded the best results with gold named entities (Table 9). In the second experiment, we incorporated the more fine-grained UMLS semantic information provided by MetaMap and attempted to find the best configuration specifically for end-to-end coreference resolution. The improvement from the first to the second experiment was greatest with the following changes to the configuration:

- SemanticCoercion(1,0) agreement method is added to the scoring function for anaphora resolution of possessive pronouns.
- The scoring function for nominal cataphora class is augmented with with SemanticType(1,1) and SemanticGroup(2,0) agreement methods. The same is applied to predicate nominative class, as well.
- HypernymList(1,1) agreement is substituted with SemanticType(2,0) and SemanticGroup(1,1) agreement methods for the appositive class.

SemanticCoercion agreement method is useful for resolving possessive pronouns, and similar methods have been proposed, mainly for gene/protein type entities [15, 17]. This agreement incorporates semantic type information for possessive pronouns, based on the headwords that they modify. For example, the anaphor *its* in the noun phrase *its absorption* is likely to refer to a substance, since substances can be absorbed; in other words, the pronoun is coerced by the headword to bear Substance semantic type. The method relies on a list of such triggers, that can indicate events that involve drugs/substances. This list consists of *effect, absorption, clearance, safety, moiety*, and *metabolism*, and is mined from the training set. SemanticType agreement method predicts agreement if the mention and the referent share semantic types, while SemanticGroup agreement predicts agreement if they share more coarse-grained semantic groups. We use UMLS semantic groups for this method [64].

The results for end-to-end coreference resolution pipeline are given in Table 11. With the improved configuration, we were able to increase F_1_ score from 49.2 to 51.7. The main improvement came from recognition of appositive links, which benefitted from using specific semantic type/group information in scoring. The F_1_ score for appositive links increased from to 43.9 to 54.3, which is only slightly lower than the F_1_ score we obtained for this class using gold entity annotations (55.3). Overall, using concept detection/normalization resulted in a relatively small drop in F_1_ score, which is not unexpected (from 53.5 to 51.7, 1.8 points). Compared to using gold entity mentions, the biggest drop involved predicate nominatives (3.5 points), while the drop was smaller for anaphora and cataphora instances, as shown in Table 11. The small drop in performance indicates that MetaMap was largely successful in accurately identifying drug/substance concepts in text.

**Table 11:** Evaluation results on the test portion of SPL coreference dataset using concept recognition/normalization with MetaMap.

	Precision	Recall	F_1_	F_1_ difference from using gold entities
*With the best configuration for gold entity mentions*				
Bio-SCoRes	61.1	41.3	49.2	
- Anaphora	61.7	43.1	50.7	
- Cataphora	66.7	28.4	39.8	
- Appositive	43.9	43.9	43.9	
- PredicateNominative	88.1	52.1	65.5	
*With the best configuration for end-to-end coreference resolution*				
Bio-SCoRes	**62.7**	**43.9**	**51.7**	**-1.8**
- Anaphora	59.6	44.9	51.2	-2.1
- Cataphora	74.0	32.4	45.1	-1.4
- Appositive	58.7	50.5	54.3	-1.0
- PredicateNominative	86.4	53.5	66.1	-3.5

Using a tool like MetaMap allows configurations that involve more fine-grained semantic information, which can be exploited by the system. SemanticType and SemanticGroup agreement methods are two such measures. We find that SemanticType and SemanticGroup methods improved performance for all classes, except anaphora. In the case of anaphora, F_1_ score dropped dramatically, from 51.2 to 27.0. This seems to be mostly due to the fact these measures favor links between mentions that share tokens (e.g., *this drug* and *the drug*), which are generally not annotated in the corpus, since they are not very informative. We also find that mapping problems might lead to errors. For example, *basis* is mapped to a concept with the semantic type Pharmacologic Substance, generating a precision error.

Another measure we experimented with is Taxonomy agreement method, which takes into account taxonomic relations in the UMLS Metathesaurus [38] and is appropriate for nominal mentions. This very fine-grained measure predicts agreement if the UMLS concept associated with the mention is an ancestor of the concept associated with the candidate referent in the UMLS concept hierarchy. This agreement is intended to capture coreference links between, for example, a drug and its class (e.g., *Cleviprex* and *this calcium channel blocker*). We predicted that this agreement would benefit identification of coreference between drugs and their classes; however, adding it led to poor performance in anaphora resolution (39.1 F_1_ score). We found that the main reason for this performance hit is that UMLS concepts that correspond to some of the reliable drug/substance hypernyms, such as *agent* and *compound*, are not in a taxonomic relationship with most of the specific drugs/substances in the UMLS. This is not entirely unexpected since such words describe a functional or a structural relationship, rather than a strict taxonomic relationship. The results involving SemanticType, SemanticGroup, and Taxonomy measures suggest that a list of high-quality hypernyms for specific semantic groups may often be adequate for good nominal coreference resolution performance.

Examining the results we obtained with the base experiment (i.e., using gold named entities), we identified errors made by different components of the framework. Errors in linguistic pre-processing were propagated to other steps of resolution. For example, we failed to identify *classes* as the head of the coordinate noun phrase *the phenylalkylamine [verapamil] and benzothiazepine [diltiazem] classes* and instead, picked *phenylalkylamine*. This resulted in a downstream precision error, since the coordinate noun phrase is then recognized as a coreferential mention; whereas, correct head identification would eliminate it according to Anaphoricity filter. Mention-referent linking, then, links the noun phrase to *BYSTOLIC* since Number and HypernymList measures both predict compatibility; the former because both *phenylalkylamine* and the referent *BYSTOLIC* are singular, and the latter because *class* is considered a drug/substance hypernym. In other cases, erroneous or unspecific dependency relations led to errors in mention detection. For example, we misidentified *either* in *either class of agents* as a distributive pronoun, since the dependency between *either* and the token it modifies, *class*, is the unspecific *dep* (dependent) relation, which we have not used in noun phrase chunking.

We observed errors in candidate referent filtering, as well. For example, Exemplification filter was unable to recognize that *spironolactone, amiloride*, and *triamterene* are exemplifications of *potassium-sparing diuretics* in the following example; therefore, the anaphora relation shown is missed and, instead, one that involves these specific drugs is created, leading to three precision errors and a single recall error. It can be argued that such anaphora relations may be acceptable to some extent, depending on the downstream task.

(11) [Potassium-sparing diuretics]_Antecedent_ (spironolactone, amiloride, triamterene, and others) or potassium supplements can increase the risk of hyperkalemia. If concomitant use of [such agents]_Anaphor_ is indicated, …

Agreement errors are also common. Number agreement is almost always required, and it predicts incompatibility between *this drug* and *Trandate tablets*, a pair annotated as coreferential. We found that absence of discourse-level semantic information is responsible for some errors. For instance, in the fragment below, *this product* is linked to the first mention of *aspirin* in the sentence, although aspirin is an ingredient of the drug, as the context of the mention makes clear.

(12) For stroke or TIA patients for whom aspirin is indicated to prevent recurrent myocardial infarction (MI) or angina pectoris, the aspirin in [this product]_Anaphor_ may not provide …

For SPLs, it would be possible to develop an agreement measure that takes into account information about the drug, such as its ingredients, brand name, and dosage, and use this information and fine-grained semantic knowledge (e.g., distinguishing substances from brand names) for a more accurate nominal coreference resolution.

Using parse tree salience to break ties between equally valid candidate referents can also lead to precision and recall errors. For example, *Lotrel therapy* is preferred to *diuretic* in the following sentence due to parse tree salience. Default salience (proximity) would have predicted the correct antecedent; however, we found that parse tree salience works better overall for personal and possessive pronouns.

(13) *In such patients, start Lotrel therapy under close medical supervision, follow closely for the first 2 weeks of treatment and whenever the dose of the benazepril component is increased or a [diuretic]_Antecedent_ is added or [its]_Anaphor_ dose increased*.

### Coreference Resolution on Discharge Summaries

Our experiments on clinical text focused on the i2b2/VA corpus [19], which was provided for training and evaluation in the 2011 i2b2 challenge on coreference resolution (available at https://www.i2b2.org/NLP/Coreference/). As mentioned earlier, the coreference annotations in this corpus are similar to those in the OntoNotes corpus, in that mention clusters, rather than mention-referent pairs, are annotated. Entity types annotated in the corpus include *problem*, *person*, *test*, and *treatment*. Pronoun mentions are also annotated, but simply as *pronoun*, and not at the fine-grained level that our framework uses. We used the portion of the corpus that did not include reports from University of Pittsburgh Medical Center (UPMC) for experiments. This portion of the dataset consists of 424 discharge summaries, of which 251 were used for training and 173 for testing. There are 3211 coreference chains annotated, with an average chain length of 4.24. The maximum chain length is 122.

The specific portion that we used formed the basis for Task 1C of the i2b2/VA challenge, which presupposed all named entity and coreferential mentions and focused on clustering these mentions. Therefore, instead of recognizing and filtering coreferential mentions as we did on the SPL dataset, we preprocessed the existing mention annotations to determine their fine-grained mention types, such as definite noun phrase or possessive pronoun. For this, we mostly used the mention detection machinery we described earlier, and augmented it with heuristics to determine the mention type based on the presence of keywords, such as *which* (for relative pronouns) and *the* (for definite noun phrases).

Domain-specific pre-processing involved segmenting the reports into sections. To determine if a line constitutes a section header, we examine its last token. If it is a colon and if the line contains at least one capitalized word, we recognize the line as a section header. With these heuristics, *DISCHARGE MEDICATIONS:*, for instance, is recognized as a section header.

We attempted coreference resolution on two types in this dataset: anaphora and, appositive. The resolution strategies we used are provided in Table 12. In this dateset, we observed that verbal mentions were sometimes annotated as coreferential; therefore, we used SemanticClass referent filtering instead of the Default filtering, which excludes verbal phrases as candidates. Table 12 shows the prominence of agreement measures based on string similarity for nominal anaphora. This is not surprising, since such agreement measures are commonly exploited in the sieve-centric approaches to coreference resolution, which generally target mention clusters, as does this task. Specifically, we implemented the following string similarity measures:

- ExactString: Two mentions are compatible, if their texts are the same, ignoring case.
- PreModifierAndHead: Two mentions are predicted to be compatible, if the substrings that consist of their pre-modifiers (excluding initial determiners and pronouns) and heads are the same, ignoring case. For instance, the mentions *a 1 cm cyst in the right lobe of the liver* and *the 1 cm cyst* are considered compatible, based on the match on *1 cm cyst*.
- RelaxedStem: This agreement measure uses Porter stemmer [65] to stem a noun phrase (excluding determiners, pronouns, and prepositions) and considers two nominal mentions as compatible if their stem overlap is greater than 50%. This measure will predict *Propofol drips* and *his propofol drip* as compatible. If the noun phrases include numbers, these numbers are expected to match strictly, to prevent a link between, for example, *70 white blood cells* and *5-10 white blood cells*.

We also implemented a more corpus-specific agreement measure called KeyValuePair, which considers as compatible two mentions, one of which is the value for the other, which is a key. We implemented this measure to account for the coreference between *Attending* and *I BUN, M.D.* in *Attending: I BUN, M.D.*, for example. This measure relies simply on the presence of a colon between mentions of the same semantic type.

**Table 12:** Configuration for coreference resolution in discharge summaries.

Mention Type	Mention Filters	Referent Filters	Agreement Methods	Post-Scoring Filters
*Anaphora resolution*				
PersonalPronoun	None	PriorDiscourse	Person(1,0)	Threshold(4)
DistributivePronoun		WindowSize(Section)	Gender(1,0)	TopScore
ReciprocalPronoun		SyntacticConfig	Animacy(1,0)	Salience(Default)
		SemanticClass	Number(1,0)	
PossessivePronoun	None	PriorDiscourse	Person(1,0)	Threshold(4)
		WindowSize(Section)	Gender(1,0)	TopScore
		SemanticClass	Animacy(1,0)	Salience(Default)
			Number(1,0)	
RelativePronoun	None	PriorDiscourse	Person(1,0)	Threshold(1)
		WindowSize(Sentence)		
		SemanticClass		
DefiniteNP	Anaphoricity	PriorDiscourse	Number(1,1)	Threshold(4)
DemonstrativeNP		WindowSize(Section)	SemanticType(2,2)	TopScore
DistributiveNP		SyntacticConfig	HeadWord(2,0)	Salience(Default)
		SemanticClass	ExactString(2,0)	
			PreModifierAndHead(2,0)	
			RelaxedStem(2,0)	
ZeroArticleNP	None	PriorDiscourse	Number(1,3)	Threshold(4)
		WindowSize(Document)	ExactString(4,0)	TopScore
		SyntacticConfig	PreModifierAndHead(4,0)	Salience(Default)
		SemanticClass	KeyValuePair(4,0)	
			RelaxedStem(3,0)	
*Appositive resolution*				
DefiniteNP	None	WindowSize(Sentence)	Number(1,1)	Threshold(4)
IndefiniteNP		SemanticClass	SyntacticAppositive(3,2)	TopScore
				Salience(Default)

In post-processing, no Mention-Referent Pair Pruning is needed. Coreference Chain Generation step involves a decision regarding whether to add the compatible mention pair to an existing cluster or to generate a new cluster. We first determine whether either mention is already clustered with other mentions. If so, the mention that is not yet clustered is added to this cluster. If both mentions are already in different clusters, these clusters are merged. If neither is clustered yet, we generate a new cluster. If a mention *m* appears between the two compatible mentions and has the same identity (i.e., its text is a substring of one of the mentions in focus and its semantic type is the same), we add mention *m* to the cluster, as well.

The domain-specific post-processing for discharge summaries is significantly more involved, since apart from zero article noun phrases, we do not generate document-level clusters in the previous steps. The first step of this phase focuses on merging coreference clusters extracted at the section level. For this, we examine every cluster and singleton within each section and determine whether they can be merged with an already formed cluster from the previous sections. To be able to merge a current cluster or singleton, *a*, with a cluster or singleton from previous sections, *b*, we stipulate that at least one of the mentions in *a* and one of the mentions in *b* are compatible based on ExactString agreement measure. The second step of this phase is concerned with the patient cluster, which often constitutes the largest cluster in the reports. In this step, all singleton mentions with the text *patient* are added to the existing patient cluster, recognized by the presence of the word *patient*. To address the cases in which the patient is referred to in second person singular (*you, your, yourself*), we also merge clusters involving these mentions to the larger patient cluster. Finally, all remaining singletons are discarded.

With the configuration given above and the post-processing steps outlined, we obtain the results in Table 13. In these experiments, we used the standard coreference evaluation metrics that were used to evaluate the systems participating in the challenge. The main evaluation criterion is the unweighted average over BCUBED, MUC, and CEAF F_1_ scores. The baseline for the task is a method that predicts all mentions to be singletons. The performance of the top ranked system in the challenge [51] is also provided.

**Table 13:** Evaluation results on the test portion of the i2b2/VA dataset.

	Precision	Recall	F_1_
Baseline	0.517	0.597	0.541
Xu et al. [51]	0.906	0.925	0.915
Bio-SCoRes	**0.838**	**0.881**	**0.858**
- BCUBED	0.964	0.944	0.954
- MUC	0.735	0.830	0.779
- CEAF	0.815	0.868	0.841
*By semantic type*			
– Test	0.796	0.700	0.735
– Person	0.816	0.903	0.850
– Problem	0.774	0.851	0.808
– Treatment	0.759	0.826	0.789
*No post-processing*			
Bio-SCoRes	0.800	0.871	0.832
- BCUBED	0.966	0.941	0.953
- MUC	0.655	0.807	0.723
- CEAF	0.777	0.869	0.821

Our results would yield a ranking that is in the lower third of the participating systems. Our system performs somewhat poorly in comparison to systems participating in the challenge; however, it should be noted that our goal in these experiments was not necessarily to get the best performance specific to this dataset, but rather to assess the generality of the framework and its adaptability. In that respect, we were able to obtain moderately successful results with some post-processing and addition of several string similarity-based agreement measures. It should also be noted that we have not incorporated any external knowledge base (e.g., Wikipedia, Probase, etc.), performed spelling correction, addressed acronyms, or attempted to distinguish between mentions of family members and patients. All these techniques were exploited by some of the better performing systems. Clearly, all these methods could be incorporated into the framework as either domain-specific pre-processing steps (e.g., spelling correction) or as agreement measures (e.g., acronyms) and this is likely to provide better performance; however, in the scope of this study, we have limited ourselves to implementing several relatively simple string similarity measures that are more crucial for mention clustering. Our pipeline performed best on person clusters, and worst on test clusters, consistent with most systems. For reference, we provide the results for individual coreference evaluation metrics, as well; we obtain the highest F_1_ score using the B-CUBED metric and the lowest with the MUC metric, again consistent with most other competition systems. We also calculated the BLANC score [12], a metric intended to address some of the shortcomings of the other metrics. We obtained an F_1_ score of 0.989 with this metric. This score is higher than that reported by the runner-up system in the i2b2 challenge (0.968) [54]. No BLANC score was reported by the top ranked system. However, since these metrics are still somewhat controversial [14], it is perhaps wise not to draw many conclusions from them.

Our results show that post-processing clearly has a positive effect on mention clustering, improving the main evaluation metric from 0.832 to 0.858. However, it is an open question whether further improvement can be obtained by enhancing the process of merging clusters/singletons, and whether we can extend the post-processing method described here to a general mention clustering method that is more central to the framework.

### Coreference Resolution on Protein Coreference Dataset

The Protein Coreference Dataset [18] has been developed to address the problem of coreference resolution in molecular biology literature (available at http://weaver.nlplab.org/~bionlp-st/BioNLP-ST/downloads/downloads.shtml). Coreference instances annotated in this dataset are mainly of anaphora type. This is due to the fact that coreference resolution is viewed as a task that supports more practical information extraction tasks, such as event extraction, in this domain. Anaphora resolution is more critical for such applications than resolution of appositive coreference. Furthermore, cataphoric instances are not common in the abstracts of biomedical articles, the text type of this dataset.

The dataset was used as the basis for a supporting task in BioNLP-ST 2011 shared task on event extraction [18]. The organizers analyze the coreferential expressions annotated in this dataset in three classes: DNP for noun phrases, RELAT for relative pronouns, PRON for other pronouns including personal, possessive and demonstrative pronouns. Few cases of other types also exist (OTHER). The best competition system achieved an F_1_ score of 34.1% [42]. In a more recent study, Nguyen et al. [17] achieved 51.3% F_1_ on the same dataset using additional semantic and discourse constraints. The F_1_ score of their system on the development portion of the dataset was found to be 62.4%.

We applied our approach to the development portion of the Protein Coreference Dataset, which consists of 150 MEDLINE abstracts. This portion of the dataset contains 463 anaphoric mentions and the distribution of these expressions by their type is as follows: RELAT (54.8%), PRON (32.2%), DNP(12.5%), OTHER (0.5%). Despite the name of the dataset, it contains anaphora relations that do not involve proteins, particularly when anaphoric mentions are relative pronouns.

The task involved recognizing anaphors and linking them to their antecedents. We used gold standard Protein entities provided with the dataset and performed our standard linguistic pre-processing, including recognition of coordinated entities. We created the resolution configuration for our experiments (shown in Table 14) using the provided training set of 500 abstracts.

**Table 14:** Configuration for the Protein Coreference Dataset.

Mention Type	Mention Filters	Referent Filters	Agreement Methods	Post-Scoring Filters
PersonalPronoun	ThirdPerson	PriorDiscourse	Person(1,0)	Threshold(4)
	PleonasticIt	WindowSize(2)	Gender(1,0)	TopScore
		SyntacticConfig	Animacy(1,0)	Salience(Parse)
		Default	Number(1,0)	
PossessivePronoun	ThirdPerson	PriorDiscourse	Person(1,0)	Threshold(4)
		WindowSize(2)	Gender(1,0)	TopScore
		Default	Animacy(1,0)	Salience(Parse)
			Number(1,0)	
			SemanticCoercion(1,0)	
DistributivePronoun	None	PriorDiscourse	Person(1,0)	Threshold(4)
ReciprocalPronoun		WindowSize(2)	Gender(1,0)	TopScore
		SyntacticConfig	Animacy(1,0)	Salience(Default)
		Default	Number(1,0)	
RelativePronoun	CoreferentialPronoun	PriorDiscourse	Adjacency(1,0)	Threshold(1)
		WindowSize(Sentence)		Salience(Default)
		Default		
DefiniteNP	Anaphoricity	PriorDiscourse	Number(1,1)	Threshold(4)
DemonstrativeNP		SyntacticConfig	HypernymList(3,0)	TopScore
DistributiveNP		Default		Salience(Default)

In this dataset, we used CoreferentialPronoun mention filter for relative pronouns to eliminate from consideration the *wh*-words that are rarely used as relative clause markers, such as *how* and *what*. We also implemented Adjacency agreement measure for relative pronoun resolution. This measure predicts compatibility if the mention and the referent are either contiguous in text or only have punctuation and prepositions between them. HypernymList and SemanticCoercion agreement methods use gene/protein word lists mined from the training set, given below. Noting that meronyms that indicate part-whole relationships can play the same role as event triggers, we used them for semantic coercion of possessive pronouns, as well.

- Hypernyms (used by HypernymList): *gene, protein, factor, cytokine, molecule, receptor, element, family*
- Meronyms (used by SemanticCoercion): *residue, domain*
- Event triggers (used by SemanticCoercion): *binding, expression, interaction, regulation, activity, localization, phosphorylation, transactivation, transcription*

Post-processing for this dataset is similar to that for SPL dataset. We generate coreference chains in the same way and, in domain-specific post-processing, remove chains that do not involve gene/proteins. No specific mention-referent pair pruning is performed.

The results on this dataset are given in Table 15. Our pipeline yields a 5-point increase in F_1_ score, compared to the method of Nguyen et al. [17], who had previously reported the best results on this dataset. Both precision and recall are improved. The fact that we have performed minimal optimization, apart from incorporating limited domain knowledge (hypernym/meronym/event trigger word lists for gene/protein entities), to achieve these results is a good indicator of the generality of our approach. One shortcoming of the dataset is that it is limited to anaphora and the majority of the coreference annotations involve relative pronouns, which are often easier to address than other types of pronouns or nominal anaphors, as confirmed with our results on the RELAT category, shown in Table 15. The system performed poorly on nominal anaphora (DNP) and moderately on pronominal anaphora (PRON). We have not attempted to resolve demonstrative pronouns, and excluding them from evaluation yields a 2.8-point increase in recall (to 49.6) and 1.2-point increase (to 51.0) in F_1_ score for the PRON class.

**Table 15:** Evaluation results on the development portion of the Protein Coreference Dataset.

	Precision	Recall	F_1_
Bio-SCoRes	**72.4**	**63.2**	**67.5**
- PRON	52.5	46.8	49.8
- RELAT	86.2	83.3	84.7
- DNP	36.4	18.5	24.5
Nguyen et al. [17]	67.8	57.8	62.4

We were somewhat concerned with the low performance on nominal anaphora; therefore, we analyzed errors involving this type more closely. We found that several anaphora instances that our pipeline identified seemed valid but were not annotated in the dataset. An example is given below. We recognized an anaphoric relation between *the cytokines* and *IL-2, IL-6, TNF-alpha, IL-10*.

(14) *We have studied the effects of prednisone (PDN), deflazacort (DFC), and dexamethasone (DXM) on the production of cytokines ([IL-2, IL-6, TNF-alpha, IL-10]_Antecedent_) by peripheral T lymphocytes, …not all [the cytokines]_Anaphor_ investigated were affected*.

On the other hand, some recall errors were due to the fact that our configuration did not consider headword agreement in scoring for nominal anaphora. There is a fair number of instances that can be resolved using head word agreement; however, we also found that such instances were not annotated consistently leading to many precision errors, the overall effect being negative. Therefore, we turned this agreement off for scoring. For example, we can identify the instance in Example (15a), if headword agreement is turned on. On the other hand, this also leads to the precision error in Example (15b), which, on the surface, seems valid but is not annotated.

(15)
  - Inhibition of transcription factors belonging to [the rel/NF-kappa B family]_Antecedent_ by a transdominant negative mutant. …The KBF1/p50 factor binds as a homodimer but can also form heterodimers with the products of other members of [the same family]_Anaphor_ …
  - Apoptosis induced through the TCR in CD4+ T cells is mostly mediated by [the inducible expression of Fas ligand (FasL)]_Antecedent_ as a primary event leading to the commitment to death. To gain a better understanding of the transcriptional events that regulate [this expression]_Anaphor_ …

Some recall errors were due to lack of number agreement, which is often a good indicator of incompatible mentions. An example is below.

(16) *However, the components of [the NF-kappaB complexes]_Antecedent_ were different in monocytes and B cells, because p50 is part of [the NF-kappaB complex]_Anaphor_ induced by CD40 triggering in both monocytes and B cells, whereas p65 was only induced in B cells*.

There were also instances of recall errors due to anaphor filtering before the linking process begins. For example, in the following sentence, the anaphor was filtered, since it is in an appositive construction with *activator protein-1 (AP-1)*. While this mention is not annotated as the referent, an earlier occurrence of *AP-1* is. Therefore, if the system were configured to identify full mention clusters, it would be able to recover this link.

(17) *…on the levels of c-Fos and Jun and on the binding and transcriptional activity of [the transcription factor]_Anaphor_, activator protein-1 (AP-1)*.

### General Remarks

Our proposed approach has several aspects that need assessment. The first aspect is the individual resolution pipelines that were implemented for different corpora and we have assessed these in previous subsections. The second aspect to assess is the smorgasbord architecture that underpins the coreference resolution configuration (i.e., resolution strategies). Our experiments demonstrate that this modular architecture is flexible and extensible. By conceptualizing coreference resolution as a set of coreferential processes (mention filtering, scoring, etc.), it allows plugging in new strategies or adapting existing strategies with relative ease. Adopting a more fine-grained view of coreference than often assumed, it also allows optimizing for certain types of coreference critical for the task at hand, while ignoring the rest. For example, anaphora of zero article noun phrases (as was performed for discharge summaries) may not be very relevant, if the goal is to extract drug-drug interactions and concept normalization is part of the pipeline. On the other hand, definite nominal anaphora is certainly critical in this case. This approach is in line with the often pragmatic nature of coreference resolution research in bioNLP.

Another aspect that needs to be assessed is the specific, rule-based baseline methods that have been implemented during the course of this study. Some of these methods are trivial, generally useful, and are not likely to require much enhancement. For example, some candidate filtering methods, such as PriorDiscourse and WindowSize, or some post-scoring functions, such as TopScore, are expected to be generally applicable. Other methods, on the other hand, provide baseline, deterministic implementations and are not necessarily optimal. This is, in particular, the case for agreement methods. For example, the baseline, naive Number agreement measure can be substituted with a probabilistic classifier trained on a specific corpus that can consider *this drug* and *Trandate tablets* as compatible, even though on the surface they are not. Some agreement methods require word lists (for example, HypernymList and SemanticCoercion) and their performance depends on the quality of these lists. For example, for our methodology to be successful on a corpus of radiology reports, it may be critical to optimize such a list of anatomical terms. Clearly, a probabilistic model can be trained to determine whether a word acts as a hypernym or not, as well. A methodology similar to what we are discussing here was followed by Rink et al. [54], who based their i2b2 coreference resolution system on the sieve architecture [20], but rather than using the deterministic sieves supplied with that method, implemented their own supervised learning-based sieve classifiers based on the provided data. Our framework also allows such optimization.

### Limitations

One aspect of the agreement measures we have not discussed at much length is the reward/penalty scores and thresholding based on the cumulative scores of candidate referents. During the course of this study, we empirically determined these values based on our intuitions and observations as well as experiments on training sets. A future enhancement may involve a more systematic selection of agreement methods and assignment of their reward/penalty scores.

As mentioned earlier, the core processing of the framework is complete once mention-referent pairs are identified, and whether to generate pairwise relations or mention clusters from these pairs is viewed as a post-processing decision. While we were able to merge mention-referent pairs into mention clusters with some degree of success in i2b2/VA discharge summaries, our results indicated that making this decision part of the core framework (for example, as a global parameter) could prove beneficial for some tasks and could improve the flexibility of our architecture further.

Certain types of coreference are not well-represented in the corpora we included in our study; for example, event anaphora (or discourse deixis) instances cases were rarely annotated. We excluded mentions that are generally used to indicate this type of coreference, such as demonstrative pronouns, from consideration. Another enhancement would be to develop filtering and agreement methods specific for this class. Recent work on discourse deixis detection and resolution [37] can be informative in this regard.

## Conclusions

We have presented a novel, highly flexible architecture for fine-grained coreference resolution of biomedical text and provided a set of strong, linguistically-based baseline methods to be used within this architecture. Experiments on several types of biomedical corpora demonstrated the extensibility of the architecture and its ease of adaptation. We were able to obtain state-of-the-art performance on a corpus focusing on biomedical literature, while our results on clinical reports indicated that the framework can be enhanced for better clustering of coreferential mentions. We also developed a new corpus of structured drug labels annotated with fine-grained coreference information, on which we trained our approach and evaluated it with success. The annotated corpus as well as the framework (Bio-SCoRes) and the specific pipelines that we implemented for evaluation are available at https://github.com/kilicogluh/Bio-SCoRes.

## Appendix

**Annotation Guidelines.** In addition to description of linguistic phenomena of interest (e.g., coreference, anaphora, etc.), the following instructions, with additional examples, were provided to annotators as guidelines:

- Annotate the coreferential mention with the entity type mention if their referent can be recovered from the rest of the text. Such expressions include:
  - Personal pronouns (e.g., *it, them, its*)
  - Demonstrative pronouns (e.g., *this, those*)
  - Relative pronouns (e.g., *which, that*)
  - Indefinite pronouns (e.g., *all*)
  - Distributive pronouns (e.g., *each, both*)
  - Reciprocal pronouns (e.g., *each other*)
  - Definite noun phrases (e.g., *the drug, the following medications*)
  - Demonstrative noun phrases (e.g., *such preparations*)
  - Indefinite noun phrases (e.g., *a vasodilator*)
  - Distributive noun phrases (e.g., *either agent*)
  - Zero article noun phrases (e.g., *medications*)
- Annotate the referent (the entity that the mention refers to) with the appropriate semantic type unless it is already pre-annotated. If there are multiple referent candidates, pick the one closest to the mention.
- Create a coreference relation between term annotations of the mention and the referent.
  - If a mention refers to multiple referents, create a separate annotation between the mention and each referent.
  - A referent can be another mention annotation.
  - Exemplifications should not be annotated.
  - If the context allows multiple interpretations for a mention, pick one that is consistent with the order in which the entities are discussed.
